# Factors related to evacuation intention when a Level 4 evacuation order was issued among people with mental health illnesses using group homes in Japan: A cross-sectional study

**DOI:** 10.1097/MD.0000000000039428

**Published:** 2024-09-06

**Authors:** Hisao Nakai, Masato Oe, Yutaka Nagayama

**Affiliations:** a Faculty of Nursing, University of Kochi, Kochi, Japan; b School of Nursing, Kanazawa Medical University, Kanazawa Medical University Hospital, Ishikawa, Japan.

**Keywords:** evacuation intention, group home, people with mental health illnesses

## Abstract

This study aimed to identify factors related to the intention to evacuate in a disaster following a Level 4 evacuation order among people with mental health illnesses living in group homes in Japan. The participants were people with mental health conditions living in group homes in Ishikawa Prefecture, Japan. We created an original self-administered questionnaire and conducted a survey among this group. Of the 625 people with mental health conditions using group homes, 152 (24.3%) responded. Of these, 110 (5.9%) who provided valid data were included in the analysis. A total of 85 (77.3%) people said that they intended to evacuate in the event of a Level 4 evacuation order. We controlled for gender, age group, type of disability, experience of participating in disaster training, fear of contracting COVID-19 during evacuation, and earthquake and flood disaster experience as confounders. After controlling for these variables, the factors associated with intention to evacuate under a Level 4 evacuation order included not having emergency food prepared (odds ratio [OR] 3.81, 95% confidence interval [CI]: 1.03–14.08); believing that group home users would help them during disasters (OR 3.08, 95% CI: 1.04–9.12); and planning to ask the local government for help (OR 2.84, 95% CI: 1.01–8.01). Group home managers should be aware that people who do not believe that other group home residents would help them, and those not wishing to seek help from local government, may decide not to evacuate. Longitudinal studies across diverse regions are needed to identify factors that affect evacuation intention.

## 1. Introduction

In recent years, climate change has caused disasters on a global scale.^[1,2]^ For example, in September 2023, heavy rains in several Mediterranean countries caused severe flooding and damage.^[3]^ In August 2023, extreme wildfires occurred in Canada. At least 17 people were killed by the fires, and more than 150,000 people were evacuated.^[4]^ In July 2023, large areas of the United States, Mexico, southern Europe, and China were hit by a heatwave, breaking high-temperature records in many regions, and resulting in casualties due to heatstroke.^[5]^ A systematic review has suggested that climate change is associated with worsening human health.^[6]^ Extreme weather and associated events such as waves, cyclones, and floods, as well as climate change-influenced events such as bushfires, continue to have a negative impact on mental health and wellbeing.^[7]^

Japan is no exception. There, climate change has caused disasters from tropical cyclones and heavy rain.^[8,9]^ In recent years, heavy rainfall has frequently occurred in Japan from early summer to autumn, resulting in serious disasters such as landslides, precipitation, and debris flows.^[10]^ This is the result of *senjyo-kousuitai*, a quasi-stationary band-shaped precipitation system, around 50 to 300 km long and 20 to 50 km wide. It is produced by successively formed and developed convective cells, lining up to organize multicell clusters, and passing or stagnating at almost the same place for a few hours, resulting in heavy rainfall.^[11]^ The damage caused by heavy rains from this phenomenon is especially serious. Extreme rains caused by *senjyo-kousuitai* in Hiroshima on August 19, 2014, caused flash floods that destroyed 330 homes and killed 75 people.^[12]^ Fourteen people died in the Kinugawa flood in September 2015.^[13–15]^ The 2017 July Northern Kyushu Torrential Rainfall Disaster caused 37 deaths and 4 people to go missing.^[16–18]^ Typhoon No. 19 in 2019 left 91 people dead and 3 missing.^[19]^ This damage from extremely heavy rain occurs almost every year in Japan. The government revised its guidelines on evacuation information in 2021, taking advantage of the revision of the Basic Act on Disaster Management. If the risk of a disaster increases, the public is provided with evacuation information based on a five-level alert system and urged to take prompt evacuation action as early as possible.^[20]^ However, there are people for whom the act of evacuation itself can be a burden. In the worst-case scenario, their lives may even be in danger. These groups include medically vulnerable people and older people living in the community. The severity and frequency of disasters increase the risk of death and injury for medically vulnerable populations.^[21]^ After the Fukushima Daiichi nuclear power plant accident following the Great East Japan Earthquake in 2011, at least 50 patients died during the mass evacuation of hospitals and nursing care facilities.^[22]^ Medically vulnerable people may not evacuate even if alert levels are increased. The intention to evacuate from hurricanes decreases with increasing age.^[23,24]^ People with low mental health and self-efficacy are also less likely to behave appropriately in response to disaster risk.^[25]^ The COVID-19 pandemic since 2020 has meant that people may be hesitant to evacuate because of fear of infection at evacuation centers.^[26,27]^ Medically vulnerable populations include people with mental health illnesses. This group is characterized by the fact that their disease or disability is not always visible. Approximately 70% of people with mental health problems have experienced difficulty in having others understand their disability. However, about half complain that it is unbearable to be looked at in a special way, to be pitied, or to be looked down upon because of their disability.^[28]^ People with mental health problems may fear that they will not receive appropriate consideration when they evacuate to a public shelter, or that they may be subject to stigma if they disclose that they have a mental health condition. Previous studies have shown that exposure to disasters and living in evacuation zones have an impact on mental health,^[29–32]^ However, no studies have investigated the intention to evacuate in the event of a disaster of people with a mental health condition who are using group homes in the community. Even if people in this group intend to evacuate, evacuation measures are an urgent issue, and this may be a problem for people who may hesitate or may not evacuate because of their mental health. Japan’s disaster alert levels state that it is desirable for all local residents to begin evacuation preparations when Level 3 evacuation information is issued, and to evacuate at Level 4. The purpose of this study was to identify the factors associated with intention to evacuate when a Level 4 evacuation order is issued among people with mental health conditions using group homes. In Japan, measures are being actively implemented to encourage deinstitutionalization of people hospitalized with mental illnesses.^[33]^ Group homes are used by people with mental health conditions who aim to live alone after being discharged from hospital, but are worried about living in the community immediately after discharge.^[34]^ It is expected that more people with mental health conditions will be living in group homes in the future. Identification of factors associated with evacuation intention when a Level 4 evacuation order is issued for this group provides evidence for appropriate evacuation behavior during disasters.

## 2. Methods

### 2.1. Target area

**Figure 1.**
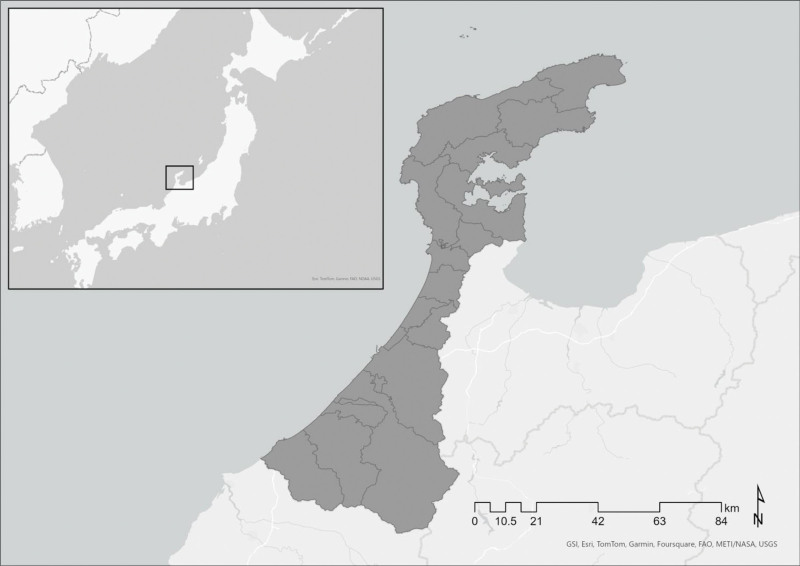
Locations in Ishikawa Prefecture, Japan.

The target area is Ishikawa Prefecture, Japan. This area is in the center of the Hokuriku region, and is long and narrow from southwest to northeast, with a coastline of approximately 584 km and an area of approximately 4186 km^2^.^[35]^ In July 2021, the population was 1127,428, of which 18,307 (3.6% of the national total) had a diagnosed mental health condition.^[36]^ Figure 1 shows the location of Ishikawa Prefecture. The map was created using Esri ArcGIS Pro 3.2.1. (ESRI, Redlands, CA).

### 2.2. Definitions

#### 2.2.1. Evacuation information by alert level

From May 2021, Japan has classified evacuation information into 5 levels, which are officially announced when appropriate. Levels 1 and 2 are warnings issued by the Japan Meteorological Agency. They mean that people should prepare for evacuation. Levels 3, 4, and 5 are evacuation orders issued by local governments. Level 3 urges vulnerable groups such as older adults to evacuate. Level 4 urges everyone to evacuate. Level 5 indicates the possibility that a disaster has already occurred and urges immediate action to ensure safety.^[20,37,38]^

### 2.3. Data collection

The participants in this study were people with mental health illnesses living in group homes in Ishikawa Prefecture. The inclusion criteria were aged 18 years or older residing in group homes in Ishikawa Prefecture, Japan; ability to communicate adequately and understand the study procedures; and willingness to provide informed consent to participate in the study. The exclusion criteria were current acute psychotic episode or other severe mental health crisis; inability to participate owing to safety or other concerns as judged by the group home personnel; unwillingness or inability to provide informed consent. We created an original questionnaire based on Japan’s “Survey on the living conditions of people with severe mental disorders after a disaster”^[39]^ and the Japan Broadcasting Corporation’s website,^[40]^ which widely publicizes considerations for vulnerable people during disasters. We used a multistage process to develop the questionnaire to ensure that it was effective. First, informed by a comprehensive literature review, we carefully generated a pool of items that captured key aspects of disaster preparedness and evacuation intentions among individuals with mental illness (e.g., disaster knowledge, experience, preparedness behaviors, and evacuation intentions). An expert panel that comprised psychiatric nurses and researchers in disaster nursing, home nursing, and psychiatric nursing then rigorously reviewed the questionnaire for validity, relevance, clarity, cultural sensitivity, and alignment with the study objectives. Finally, to assess comprehensiveness, clarity, and ease of administration, we pilot-tested the questionnaire with a separate group of researchers. The results of this pilot test informed further refinement of the items and response options to ensure optimal data collection (see Questionnaire, Supplemental Digital Content, http://links.lww.com/MD/N535). Questionnaires were sent to 306 group homes in Ishikawa Prefecture,^[41]^ and group home staff asked participants to respond. Participants were asked to choose between a web-based survey accessible via a QR code or a paper-based survey using a survey form. All surveys were anonymous and self-administered. The web-based survey was generated using SurveyMonkey, a cloud-based survey development application.

In a letter to participants, we explained the purpose and significance of the study, the research method, that participation in the study was voluntary, that participants’ responses were anonymous, and that individuals would not be identified even if they responded to the questionnaire. This study was conducted from December 15, 2022 to January 27, 2023.

### 2.4. Survey content

#### 2.4.1. Background of people with mental health illnesses using group homes

We asked participants to provide their gender, age, type of disability (mental disability, physical disability, intellectual disability).

#### 2.4.2. Experience of being affected by a disaster

We asked respondents whether they had experience of being affected by a disaster, with answer options being “No” or “Yes.” The items were: tsunami, landslide, fire, flood, wind, heavy rain, typhoon, heavy snowfall, and earthquake.

#### 2.4.3. Disaster preparedness

We asked about disaster preparedness, with each question having possible responses of “No” or “Yes.” The questions were whether participants had prepared water, emergency food, toilet paper, tissue (Kleenex), matches, and a portable stove. We also asked whether participants had experience of participating in disaster training, with possible answers of “No”‘ or “Yes.”

#### 2.4.4. Plans about how to manage in a disaster

We asked about participants’ plans about how they would manage in a disaster. These questions all had 4 possible responses: disagree, somewhat disagree, slightly agree, and agree. The items were: I’m planning to ask a friend for help, I’m planning to ask the family doctor for help, I’m planning to ask the local government for help, I’m planning to ask the hospital staff for help, I don’t care what happens if there is a disaster. The items on help during a disaster were as follows: Group home users would help me during a disaster, The counselor at the group home would help me during a disaster, The local people would help me during a disaster. The item on fear of COVID-19 was I’m afraid of contracting COVID-19 during evacuation.

### 2.5. Analytical methods

Among respondents who provided valid data on gender and age, only those whose disability was categorized as mental disability were included in the analysis. Data were analyzed for respondents who answered all items on experience of disasters, disaster preparedness, and disaster response planning. A total of 110 respondents who met these criteria were included in the analysis. The mean and standard deviation of the age of the analyzed participants were calculated. For the age group, we calculated the distribution by 10s (10s–80s) and classified people as under 65 and over 65 years old. We classified the type of disability as >1 (that is, physical disability or intellectual disability as well as mental disability) or just mental disability. For disaster planning items, the responses for “disagree” and “somewhat disagree” were combined into “disagree” and the responses for “slightly agree” and “agree” were combined into “agree.” To examine the relationship between an affirmative response to the statement “I intend to evacuate when a Level 4 evacuation order is issued” and each item, the χ^2^ test or Fisher exact test were used. Binomial logistic regression analysis was used to assess factors associated with the intention to evacuate when a Level 4 evacuation order is issued. Intention to evacuate when a Level 4 evacuation order is issued was the dependent variable. The covariates were age group, gender, type of disability, experience of participating in disaster training, fear of contracting COVID-19 during evacuation, and experience of earthquakes^[42]^ and floods.^[11]^ All variables for which a statistically significant association (*P* < .05) was found in univariate analysis were force-entered. All variables were entered after checking for multicollinearity (variance inflation factor ≥ 10). The significance level was set at 5%. IBM SPSS version 27 (IBM Corp., Armonk, NY) was used for all statistical analyses.

## 3. Ethical considerations

This study was carried out with the approval of the Kanazawa Medical University Medical Research Ethics Review Committees at the authors’ universities (No. I765). The participants were given a written informed consent form and were informed of the purpose and importance of the study, the survey method, the fact that participation was voluntary, and the fact that they would not be personally identified when the results were made public. Participants completed a self-administered questionnaire. Completion of the questionnaire implied their consent.

## 4. Results

### 4.1. Overview of people with mental health illnesses using group homes

We asked 1857 people in 306 group homes to complete the survey and received responses from 234 (12.6%). Of these, 214 used the paper-based survey form and 20 the web-based survey form. Overall, 110 (5.9%) responses were suitable for analysis, of whom 72 (60.5%) were from men and 38 (31.9%) from women. The mean age (standard deviation) was 50.9 years (15.2), with 29 (24.4%) in their 50s and 23 (19.3%) in their 60s. In total, 97 people (81.5%) had only a mental disability, and 5 people (4.2%) had both a mental disability and an intellectual disability (Table 1).

**Table 1:** Background of people with mental health conditions using group homes N = 110.

Item	Category			Intention to evacuate when a Level 4 evacuation order is issued				*P* value	
		Total		No		Yes			
		n	%	n	%	n	%		
*Background of people with mental health illnesses using group homes*									
Sex	Men	72	60.5	18	25.0	54	75.0	0.434	
	Women	38	31.9	7	18.4	31	81.6		
Age, mean (standard deviation), years	50.9 (15.2)	110	92.4	25	22.7	85	77.3	0.554	
	10s	2	1.7	2	100.0	0	0.0		
	20s	11	9.2	3	27.3	8	72.7		
	30s	15	12.6	3	20.0	12	80.0		
	40s	18	15.1	3	16.7	15	83.3		
	50s	29	24.4	5	17.2	24	82.8		
	60s	23	19.3	6	26.1	17	73.9		
	70s	11	9.2	3	27.3	8	72.7		
	80s	1	0.8	0	0.0	1	100.0		
Age group	Under 65 years old	85	71.4	19	22.4	66	77.6	0.863	
	65 years old or more	25	21.0	6	24.0	19	76.0		
Type of disability	More than 1 type (mental disability plus physical disability and/or intellectual disability)	13	10.9	2	15.4	11	84.6	0.729	a
	Mental plus intellectual disability	5	4.2	0	0.0	5	100.0		
	Mental plus physical disability	7	5.9	2	28.6	5	71.4		
	All 3	1	0.8	0	0.0	1	100.0		
	Mental disability only	97	81.5	23	23.7	74	76.3		

χ^2^ test except a: Fisher exact test.

### 4.2. Experience and preparation for disasters

Overall, 28 people (25.5%) had experienced earthquakes, and 19 people (17.3%) had experienced each of typhoons and heavy snowfall. A total of 92 people (77.3%) had experience of participating in disaster training. Apart from the usual stock of supplies, 18 people (15.1%) each had water and tissues (kleenex), and 15 people (12.6%) had tissues (kleenex). A total of 28 people (23.5%) said that they did not know what preparations to make. Overall, 85 people (77.3%) said they would evacuate if a Level 4 evacuation order was issued, and 25 people (22.7%) would not do so (Table 2).

**Table 2:** Disaster preparedness, plans for managing in a disaster and evacuation intention when there is a Level 4 evacuation order among survey respondents. N = 110.

Item				Intention to evacuate when a Level 4 evacuation order is issued				*P* value	
	Category	Total		No		Yes			
		n	%	n	%	n	%		
*Disaster preparedness* (*multiple answers*)									
Experience of participating in disaster training	No	18	15.1	5	27.8	13	72.2	.552	a
	Yes	92	77.3	20	21.7	72	78.3		
*Have stores of*:									
Water	No	92	77.3	19	20.7	73	79.3	.236	a
	Yes	18	15.1	6	33.3	12	66.7		
Emergency food	No	95	79.8	18	18.9	77	81.1	.040	a
	Yes	15	12.6	7	46.7	8	53.3		
Toilet paper	No	94	79.0	21	22.3	73	77.7	.757	a
	Yes	16	13.4	4	25.0	12	75.0		
Tissue (Kleenex)	No	92	77.3	21	22.8	71	77.2	>.99	a
	Yes	18	15.1	4	22.2	14	77.8		
Matches	No	105	88.2	23	21.9	82	78.1	.318	a
	Yes	5	4.2	2	40.0	3	60.0		
Potable stove	No	104	87.4	22	21.2	82	78.8	.129	a
	Yes	6	5.0	3	50.0	3	50.0		
I don’t know what preparations to make	No	82	68.9	18	22.0	64	78.0	.740	
	Yes	28	23.5	7	25.0	21	75.0		
*Planning for a disaster* (*multiple answers*)									
I’m planning to ask a friend for help.	Disagree	75	63.0	21	28.0	54	72.0	.053	
	Agree	35	29.4	4	11.4	31	88.6		
I’m planning to ask the family doctor for help	Disagree	38	31.9	12	31.6	26	68.4	.108	
	Agree	72	60.5	13	18.1	59	81.9		
I’m planning to ask the local government for help	Disagree	39	32.8	14	35.9	25	64.1	.015	
	Agree	71	59.7	11	15.5	60	84.5		
I’m planning to ask the hospital staff for help	Disagree	46	38.7	11	23.9	35	76.1	.801	
	Agree	64	53.8	14	21.9	50	78.1		
Group home users will help me during a disaster	Disagree	59	49.6	18	30.5	41	69.5	.036	
	Agree	51	42.9	7	13.7	44	86.3		
The counselor at the group home will help me during a disaster	Disagree	32	26.9	10	31.3	22	68.8	.172	
	Agree	78	65.5	15	19.2	63	80.8		
The local people will help me during a disaster	Disagree	78	65.5	18	23.1	60	76.9	.891	
	Agree	32	26.9	7	21.9	25	78.1		
I don’t care what happens if there is a disaster.	Disagree	94	79.0	20	21.3	74	78.7	.328	a
	Agree	15	12.6	5	33.3	10	66.7		
I’m afraid of contracting COVID-19 during evacuation	Disagree	27	22.7	10	37.0	17	63.0	.041	
	Agree	83	69.7	15	18.1	68	81.9		

χ^2^ test except a: Fisher exact test.

### 4.3. Planning for a disaster

Overall, 83 people (69.7%) said they were afraid of contracting COVID-19 during evacuation. A total of 78 people (65.5%) said local people would help them during a disaster, 72 people (60.5%) were planning to ask the family doctor for help, and 71 people (59.7%) were planning to ask the local government for help (Table 2).

### 4.4. Factors related to evacuation intention in a Level 4 alert

The following variables were significantly associated with evacuation intention when a Level 4 alert is issued: Not having emergency food prepared (n = 77, 81.1%; *P* = .040), planning to ask the local government for help (n = 60, 84.5%; *P* = .015), expecting group home users to help them during a disaster (n = 44, 86.3%; *P* = .036), and being afraid of contracting COVID-19 during evacuation (n = 68, 81.9 %; *P* = .041) (Table 2).

After controlling for the effects of gender, age group, type of disability, fear of contracting COVID-19 during evacuation, experience of participating in disaster training, and previous experience of earthquakes or floods, there were 3 factors associated with probability of evacuation. Those who had no emergency food stores were 3.81 times more likely to evacuate than those who had stored food (odds ratio [OR] 3.81, 95% confidence interval [CI]: 1.03–14.08). Those who expected other group home users to help them during disasters were 3.08 times more likely to evacuate than those who did not expect this help (OR 3.08, 95% CI: 1.04–9.12). Finally, those who planned to ask the local government for help were 2.84 times (OR 2.84, 95% CI: 1.01–8.01) more likely to evacuate than those who did not have this plan (Table 3).

**Table 3:** Factors related to evacuation intention when a Level 4 evacuation order is issued. N = 110.

Item	Category	OR	95% CI		*P* value
			Lower limit	Upper limit	
Sex	Women/men	0.88	0.25	3.05	.837
Age group	65 years old or more/under 65 years old	1.08	0.33	3.52	.904
Type of disability	Mental disability/more than 1 type of disability	0.79	0.14	4.30	.780
Afraid of contracting COVID-19 during evacuation	Yes/no	2.66	0.86	8.24	.089
Experience of participating in disaster training	Yes/no	1.24	0.30	5.15	.769
Earthquake damage experience	Yes/no	1.33	0.38	4.71	.656
Flood disaster experience	Yes/no	0.18	0.02	1.60	.124
Emergency food preparation	No/yes	3.81	1.03	14.08	.045
Group home users will help me in a disaster	Agree/disagree	3.08	1.04	9.12	.042
I’m planning to ask the local government for help	Agree/disagree	2.84	1.01	8.01	.049

Binomial logistic regression analysis.

CI = confidence interval, OR = odds ratio.

## 5. Discussion

We investigated the disaster experience and plans for disaster among people with mental health conditions living in group homes in Ishikawa, Japan, together with factors associated with intention to evacuation if a Level 4 evacuation order is issued. According to a survey by the Ministry of Health, Labor and Welfare in 2021, 60.0% of group home users nationwide were male, with 23.1% in their 50s, and 22.9% in their 40s, 74.4% having an intellectual disability, and 53.1% having a mental health problem.^[43]^ Among our study participants, 60.5% were male and 24.4% were in their 50s, with the second largest group in their 60s. The difference in age from the national survey may be because this study targeted people with mental health illnesses living in group homes, and included people with both intellectual and physical disabilities. Previous reports have shown that older people are less likely to evacuate,^[23,24,44]^ and the aging of group home users may therefore be a concern when planning evacuation during disasters.

Overall, 20.8% of the world’s earthquakes of magnitude 6 or higher occur in Japan, which is extremely high considering that Japan’s land area is just 0.25% of the world.^[45]^ Ishikawa Prefecture, the target area of this research, is located almost in the center of Japan on the Japan Sea side and has a climate with short sunshine hours, heavy rain, and snow in the winter.^[46,47]^ Since 2000, heavy snowfall has frequently occurred in Ishikawa and surrounding prefectures, causing severe damage.^[48]^ Overall, 28 of our study participants (25.5%) had experienced earthquakes, 19 people (17.3%) had experience of each of heavy snow and typhoons, and 15 people (13.6%) had experience of heavy rain. This result may have been influenced by the number of earthquakes in Japan, the regional characteristics of the target area, and damage caused by heavy snowfall in recent years.

Even considering the background of study participants, their fear of contracting COVID-19 during evacuation, experience of participating in disaster training, and previous experience of earthquakes and floods, people who said they did not have emergency food, thought that other group home users would help them during a disaster, or were planning to ask the local government for help were more likely to evacuate if a Level 4 evacuation order is issued. This means that people who do not expect help from others in the group home, or who have no plans to ask the local government for help are most likely to remain in group homes after a Level 4 evacuation order has been issued and the local government has strongly urged people to evacuate. The frequent occurrence of extremely heavy rains caused by Senjyo-Kousuitai in Japan in recent years^[10,11]^ has meant that local people have had more opportunities to see evacuation life in the media and social networking services.^[49]^ People with mental health illnesses living in group homes may therefore not have emergency food prepared because they think they will easily be able to obtain this at an evacuation center. However, this is speculation at this stage, and should be investigated further in future.

It has been reported that medically vulnerable people, such as people using ventilators, are less willing to evacuate than the general population.^[50–52]^ In Japan, during and after the Great Hanshin-Awaji Earthquake of 1995, the Great East Japan Earthquake of 2011, and the Kumamoto Earthquake of 2016, medically vulnerable children and their families were likely to avoid evacuating to public shelters because it would cause a nuisance to others and be difficult to continue care because of the lack of private space.^[53–55]^ In addition to the threat posed by the COVID-19 pandemic, health disparities due to socioeconomic inequality and medical vulnerability are known to impede evacuation in the event of hurricanes.^[27,52]^ People with mental health conditions may experience stigma from others in normal times,^[56,57]^ but measures must be taken to allow group home users to leave the danger area in a timely manner when a Level 4 evacuation order is issued. With 30.5% of our respondents indicating that they do not believe that group home users would help them in the event of a disaster and 35.9% indicating that they would not seek assistance from local authorities in the event of a disaster, these people are likely to stay in their group homes instead of evacuating if threatened by a disaster. Many mental disabilities are so-called “invisible disabilities,”^[28]^ and people with these conditions may therefore may not receive appropriate support without declaring their disability. It is therefore important to ensure that people with mental health conditions—as well as those with physical disabilities or users of medical devices—feel comfortable evacuating to public shelters.

This study had some limitations. We did not use a formal sample size calculation. This may limit the generalizability of the findings to other settings and populations. The use of sample size calculations is desirable in future studies to ensure the statistical representativeness of the sample. Furthermore, the low response rate requires caution when interpreting the present findings. Although the survey was distributed to group residents by the group home support personnel, some residents may have been unable to respond without individual support. The development of more rigorous research methods is needed in future research on this population.

The population was limited to people with mental health conditions using group homes in Ishikawa Prefecture, Japan. We asked participants to self-report their disability type; however, we have been unable to confirm the reliability of their responses regarding disability. The timing of the survey, such as whether it was in a season when disasters occur frequently, may have influenced the survey results. The study findings therefore cannot be generalized. People who had only recently started living in a group home may also have responded without fully considering possible disasters and evacuation plans. This study was cross-sectional in design and it is therefore not possible to establish a causal relationship between the variables under investigation.

## 6. Conclusions

There have been a large number of Level 4 evacuation orders issued because of severe heavy rain related to climate change in recent years. We therefore recommend that group home managers thoroughly discuss disaster preparedness and evacuation actions with people with mental health conditions and other residents. In particular, they should be aware that people who do not think that group home residents will help them or who do not want to ask the local government for help may decide not to evacuate. People with mental health illnesses who intend to evacuate when a Level 4 evacuation order is issued may not make any preparations, thinking that emergency food will be available at evacuation centers. We assume that those expecting help from other residents, or planning to ask the local government for help, have a good relationship with other residents and a high level of trust in the local government, but we did not ask about this. It is possible that preparation of emergency food, and expectations about help from other group home residents and local government may be related to evacuation behavior during disasters among group home users with mental health conditions. However, the cross-sectional design of this study makes it difficult to infer any causal relationships. Longitudinal studies across diverse regions and populations are needed to confirm these findings and identify long-term influences on evacuation intentions.

## Acknowledgments

We thank Melissa Leffler, MBA, from Edanz (https://jp.edanz.com/ac) for editing a draft of this manuscript.

## Author contributions

**Conceptualization:** Hisao Nakai, Masato Oe, Yutaka Nagayama.

**Data curation:** Hisao Nakai, Masato Oe, Yutaka Nagayama.

**Formal analysis:** Hisao Nakai, Masato Oe.

**Funding acquisition:** Hisao Nakai.

**Investigation:** Hisao Nakai, Masato Oe, Yutaka Nagayama.

**Methodology:** Hisao Nakai, Masato Oe, Yutaka Nagayama.

**Project administration:** Hisao Nakai.

**Resources:** Hisao Nakai, Masato Oe, Yutaka Nagayama.

**Supervision:** Hisao Nakai.

**Validation:** Hisao Nakai, Masato Oe.

**Visualization:** Hisao Nakai.

**Writing – original draft:** Hisao Nakai, Masato Oe, Yutaka Nagayama.

**Writing – review & editing:** Hisao Nakai, Masato Oe, Yutaka Nagayama.

## Supplementary Material


