# Supplementary material for: Factors related to evacuation intention when a Level 4 evacuation order was issued among people with mental health illnesses using group homes in Japan: A cross-sectional study
Source: Medicine (Baltimore). 2024 Sep 6;103(36):e39428. doi: 10.1097/MD.0000000000039428 (PMC11384056; doi:10.1097/MD.0000000000039428)
Supplement: Supplementary file 1 [file medi-103-e39428-s001.docx]

**Survey on Disaster Preparedness, Knowledge, and Evacuation Intention among Group Home Residents**

1. What is your gender? (Man, Woman)
2. What is your age?
3. Please select all the disabilities that apply to you:

mental disability, physical disability, intellectual disability

1. Please select which of the following classifications apply to your level of disability:

Classification: 1, 2, 3, 4, 5, 6, Not Applicable, I Don’t Know

1. Where were you living before you entered your current group home?

Hospital, Another group home, Private residence, Other (please specify):

1. Are you currently using any health, welfare, or nursing care services? Please select all the services you are using.

Do not use any services, Daycare services (e.g., daycare centers, day services), Home-based services (e.g., home nursing, home helpers), Employment services (e.g., sheltered workshops, employment support services), Other (please specify):

1. Are you currently employed? (No, Yes)
2. How often do you socialize with your neighbors? (Never, Rarely, Sometimes, Often)
3. Do you socialize with other residents of the group home? (Never, Rarely, Sometimes, Often)

Next, we would like to ask you about disasters.

1. Have you ever been affected by a natural disaster? (No, Yes)
2. Please select all the natural disasters that you have been affected by: Tsunami, Landslide, Fire, Flood, Wind, Heavy rain, Typhoon, Heavy snowfall, Earthquake, Other (please specify):
3. Do you have any emergency supplies prepared in addition to your regular household items?

No (Skip to Question 14), Yes (Go to Question 13)

1. Please select all of the emergency supplies you have prepared: Water, Emergency food, Toilet paper, Tissue (Kleenex), Matches, Portable stove, Other (please specify):
2. Please select all the reasons why you do not have emergency supplies prepared: I am unsure of what supplies to prepare, I do not believe that any natural disasters will occur in my area, I find it cumbersome to prepare emergency supplies, The cost of preparing emergency supplies is too high, I doubt the effectiveness of emergency preparedness measures, I do not feel anxious about the possibility of a disaster, I do not have a place to store emergency supplies, I am unable to manage the upkeep of emergency supplies, Other (please specify):
3. Please select all the natural disasters that you associate with your area: Earthquake, Tsunami, Typhoon, Heavy rain, Strong winds, River flooding, Heavy snowfall, Landslides, Fire, Other (please specify):
4. How well do you know the tsunami risk in your area? How well do you know the flood risk in your area? How well do you know the landslide risk in your area? (I don’t know, I don’t know much, I have some knowledge, I am well-informed)
5. Do you know the location of the nearest general evacuation shelter? Have you ever been to a general evacuation shelter? Do you know the location of the nearest welfare evacuation shelter? Have you ever been to a welfare evacuation shelter? (No, Yes)
6. Please select the statement that best reflects your opinions about evacuation shelters: I am eligible to use a general evacuation shelter, I would evacuate to a general evacuation shelter if it were open, I am eligible to use a welfare evacuation shelter, I would evacuate to a welfare evacuation shelter if it were open. (I don’t think so, I’m not sure, I think so to some extent, I think so)
7. Do you think you will receive help if you become a victim of a disaster? Family, Friends, Group home users, Counselor at the group home, Local people, Group home staff, Hospital staff. (I don’t think so, I’m not sure, I think so to some extent, I think so)
8. Please select all the information and communication devices that you use: Smartphone, Feature phone, Personal computer, Tablet, Group home landline phone, Other (please specify):
9. Please select all the sources of information that you would rely on to make a decision about whether or not to evacuate during an emergency: Television, Radio, Smartphone, Feature phone, Personal computer, Local government officials, Group home staff, Neighbors, Other (please specify):
10. Would you evacuate if an Evacuation of the Elderly, Etc. (Level 3) warning was issued? (No, Yes (Skip to Question 24))
11. If you would not evacuate in the event of an Evacuation Order Level 3 (Evacuation of the Elderly, Etc.), please select the reason that best reflects your decision: I believe I am safer staying in the group home, I am concerned about the safety of traveling to an evacuation shelter, I am unsure if my neighbors would be evacuating, Other (please specify):
12. If an Evacuation Order Evacuation Instruction (Level 4) was issued, would you evacuate? No, Yes (Skip to Question 26)
13. If you would not evacuate in the event of an Evacuation Order Level 4 (Evacuation Instruction), please select the reason that best reflects your decision: I believe I am safer staying in the group home, I am concerned about the safety of traveling to an evacuation shelter, I am unsure if my neighbors would be evacuating, Other (please specify):
14. If an Evacuation Order Emergency Safety Measures (Level 5) was issued, would you evacuate? No, Yes (Skip to Question 28)
15. If you would not evacuate in the event of an Evacuation Order Level 4 (Emergency Safety Measures), please select the reason that best reflects your decision: I believe I am safer staying in the group home, I am concerned about the safety of traveling to an evacuation shelter, I am unsure if my neighbors would be evacuating, Other (please specify):

Next, we would like to ask you about evacuation.

1. Have you ever participated in disaster training before? (No, Yes)
2. Would you be interested in participating in a disaster drill in the future if you were invited? (No, Yes, Unsure)
3. Please select the option that best reflects your opinion: (I don’t think so, I’m not sure, I think so to some extent, I think so)
   1. I can imagine myself living in a public evacuation center (like a gymnasium or community center) during a disaster.
   2. I don’t care what happens if there is a disaster.
   3. I would prefer to be left alone during a disaster.
   4. I am unable to stay in crowded places.
   5. I do not want to leave my current living space.
   6. I’m planning to ask the family doctor for help.
   7. I’m planning to ask the local government for help.
   8. I would seek help from the facility I attend.
   9. I would seek help from friends.
   10. I need my own personal space during evacuation.
   11. I am concerned about interpersonal relationships at the evacuation site.
   12. I am concerned about public stigma.
   13. I believe I can live in a public shelter.
4. How long do you think you could live in a public shelter (e.g., gymnasium or community center) during a disaster?

(Not at all, A few hours, Half a day, 1 day, 2 days, 3 days, 4 days, 5 days, 6 days, 7 days, 8 days or longer)

1. Would you disclose your medical condition to others during an evacuation? (No, Yes (Skip to Question 34), Other (please specify):
2. Please explain why you would not disclose your medical condition to others during an evacuation:
3. Are you afraid of COVID-19 during evacuation? (I don’t think so, I’m not sure, I think so to some extent, I think so)
4. What is your biggest concern during a disaster?

Thank you for participating in the survey.
